# Draft Whole-Genome Sequences of *Zhihengliuella halotolerans* La12 and *Microbacterium kitamiense* Sa12, Strains with Cellulase Activity, Isolated from the Qinghai-Tibetan Plateau

**DOI:** 10.1128/genomeA.01531-17

**Published:** 2018-02-08

**Authors:** Mingming Chen, Na Qin, Wenqin Pei, Qianhui Li, Qing Yang, Yao Chen, Duntao Huang, Yingying Xiang, Ling Lin

**Affiliations:** a Provincial Key Laboratory of the Conservation and Exploitation Research of Biological Resources, College of Life Sciences, Anhui Normal University, Wuhu, Anhui, People’s Republic of China

## Abstract

We report the complete genome sequences of cellulolytic strains *Zhihengliuella halotolerans* La12 and *Microbacterium kitamiense* Sa12, which were isolated from soil samples collected from the Qinghai-Tibetan Plateau in Western China. The final assemblies of La12 and Sa12 comprise 3,712,694 bp, with over 111 contigs, and 3,830,439 bp, with over 39 contigs, respectively.

## GENOME ANNOUNCEMENT

Cellulose, as the most abundant renewable resource, has an important role in solving the problem of resource shortages. Cellulose can also be degraded into polysaccharides by three kinds of cellulase under simultaneous hydrolysis processes. Until now, many cellulolytic bacteria have been found and proven to produce efficient cellulases, which serve as very good candidates for use in paper, food, and bioenergy industrial applications. To satisfy industrial requirements, however, more study is needed to isolate hyperproducers with high pH stability and good tolerance to a wide variety of deleterious chemicals. The strain La12 was isolated from a wetland soil sample collected from Pulan, Tibet, Western China. In the phylogenetic dendrogram based on 16S rRNA gene sequence analysis, this strain formed a separate clade next to the genera *Micrococcus* and *Citricoccus* within the family *Micrococcaceae* (1). The strain Sa12 was isolated from a wasteland soil sample in Saga, Tibet, Western China. According to the homologous analysis results and the system tree, the strain species was determined to be *Microbacterium kitamiense* (2). It has been shown that strains La12 and Sa12 are salt-tolerant strains capable of breaking down cellulose and producing polysaccharide.

In the present study, the complete genome sequences of strains La12 and Sa12 were determined with whole-genome shotgun sequencing by using Illumina technology. These sequences were quality processed and trimmed, and all quality-controlled reads were assembled using Velvet (3) software. For gene prediction, the software tool Prodigal (4) was used. Genes coding for rRNA and tRNA were identified using tRNAscan-SE (5) and RNAmmer (6). The genome size of La12 is 3,712,694 bp with a 68.43% G+C content, 111 contigs, and 3,545 predicted genes (including 3,491 coding sequences [CDSs]). Two paired-end libraries were sequenced, producing 5,328,392 raw reads. In addition, 3 tRNA-coding genes and 51 rRNA-coding genes were also found, and the total assembly length was 3.71 Mb.

The cluster of orthologous groups (COG) analysis of La12 showed that 258 genes were related to carbohydrate transport and metabolism, with 114 involved in central carbohydrate metabolism, including amino acid metabolism (17 genes), lipid metabolism (8 genes), and polysaccharide metabolic processes (5 genes). A deeper study of some of the genes predicted to be associated with glycoside hydrolase will be performed to evaluate the functional activity and characteristics of the cellulase from *Zhihengliuella halotolerans* La12.

Sa12 has a genome size of 3,830,439 bp with G+C content of 68.15%, 39 contigs, and 3,715 predicted genes (including 3,666 coding sequences [CDSs]). Two paired-end libraries were sequenced, producing 5,126,728 raw reads. The overall G+C content of the chromosome was 66.99%. In addition, 44 tRNA-coding genes and 5 rRNA-coding genes were also found, and the total assembly length was 3.65 Mb.

The clusters of orthologous groups (COG) analysis of Sa12 showed that 258 genes were related to carbohydrate transport and metabolism, with 138 belonging to central carbohydrate metabolism, including amino acid metabolism (52 genes), lipid metabolism (20 genes), and polysaccharide metabolic processes (8 genes). A deeper study of the genes predicted to be associated with endoglucanases will be performed to evaluate the functional activities and characteristics of the cellulase from *Microbacterium kitamiense* Sa12.

### Accession number(s).

This whole-genome shotgun project has been deposited at GenBank under the accession no. PGGT00000000 (*Zhihengliuella halotolerans* La12) and PGGU00000000 (*Microbacterium kitamiense* Sa12). The versions described in this paper are PGGT01000000 and PGGU01000000, respectively.
